# Rapamycin doses sufficient to extend lifespan do not compromise muscle mitochondrial content or endurance

**DOI:** 10.18632/aging.100576

**Published:** 2013-07-16

**Authors:** Lan Ye, Anne L. Widlund, Carrie A. Sims, Dudley W. Lamming, Yuxia Guan, James G. Davis, David M. Sabatini, David E. Harrison, Ole Vang, Joseph A. Baur

**Affiliations:** ^1^ State Key Laboratory of Reproductive Medicine, Nanjing Medical University, Nanjing, China; ^2^ Institute for Diabetes, Obesity, and Metabolism and Department of Physiology, Perelman School of Medicine, University of Pennsylvania, Philadelphia PA 19104, USA; ^3^ Department of Science, Systems and Models, Roskilde University, Roskilde, Denmark; ^4^ Division of Trauma, Critical Care, and Emergency Surgery, University of Pennsylvania, Philadelphia PA 19104, USA; ^5^ Whitehead Institute for Biomedical Research, Cambridge MA 02142; Department of Biology, MIT, Cambridge, MA 02139; Howard Hughes Medical Institute, MIT, Cambridge, MA 02139; Broad Institute of Harvard and MIT, Seven Cambridge Center, Cambridge, MA 02142; The David H. Koch Institute for Integrative Cancer Research at MIT, Cambridge, MA 02139, USA; ^6^ The Jackson Laboratory, Bar Harbor, ME 04609, USA

**Keywords:** Biogenesis, longevity, endurance, sarcopenia, mTOR, PGC-1alpha

## Abstract

Rapamycin extends lifespan in mice, but can have a number of undesirable effects that may ultimately limit its utility in humans. The canonical target of rapamycin, and the one thought to account for its effects on lifespan, is the mammalian/mechanistic target of rapamycin, complex 1 (mTORC1). We have previously shown that at least some of the detrimental side effects of rapamycin are due to “off target” disruption of mTORC2, suggesting they could be avoided by more specific targeting of mTORC1. However, mTORC1 inhibition *per se* can reduce the mRNA expression of mitochondrial genes and compromise the function of mitochondria in cultured muscle cells, implying that defects in bioenergetics might be an unavoidable consequence of targeting mTORC1 *in vivo*. Therefore, we tested whether rapamycin, at the same doses used to extend lifespan, affects mitochondrial function in skeletal muscle. While mitochondrial transcripts were decreased, particularly in the highly oxidative soleus muscle, we found no consistent change in mitochondrial DNA or protein levels. In agreement with the lack of change in mitochondrial components, rapamycin-treated mice had endurance equivalent to that of untreated controls, and isolated, permeabilized muscle fibers displayed similar rates of oxygen consumption. We conclude that the doses of rapamycin required to extend life do not cause overt mitochondrial dysfunction in skeletal muscle.

## INTRODUCTION

Aging is the most important risk factor for morbidity and mortality in Western society today. Due to the parallel rise in the risk for many different conditions, individuals often present with multiple comorbidities, and there is a limit to the benefit that can be obtained through therapies for any individual disease. For example, it has been estimated that a complete cure for cancer would extend the average human lifespan by about 3 years [1]. On the other hand, reducing calorie intake by ~40% while maintaining adequate nutrition slows the progression of most age-related changes simultaneously and extends life by 30-50% in rodents [2, 3]. Unfortunately, dietary restriction (DR) has major limitations as an approach to improve human health and longevity. First, it is likely that many would be unwilling or unable to maintain the requisite lifestyle [4]. Second, the regimen must be started early in life to obtain the maximal benefit [5-7]. Finally, studies in primates have yielded conflicting results. While there is general agreement that DR improves health and decreases age-related diseases, only one of the two ongoing studies has demonstrated an effect on overall survival [6, 8]. Identifying new, more generally applicable ways to target the aging process is an important goal for gerontology, and a promising approach to the prevention and treatment of age-related diseases.

Rapamycin, an inhibitor of the mammalian/mechanistic target of rapamycin (mTOR), presents a tantalizing possibility for a longevity drug [9]. It is the only compound that has extended both mean and maximum lifespan in both genders of mice by the rigorous standards of the National Institute on Aging's Intervention Testing Program [10, 11], and has been shown to slow the progression of multiple age-related phenotypes in mice [12-16]. Rapamycin works even when treatment is delayed until 20 months of age (equivalent to ~60 years for a human), and would not require any dietary modification. Because rapamycin has been used clinically as an immunosuppressant and chemotherapeutic, there is an extensive body of literature documenting its tolerability and side effects [17]. Rapamycin increases the risk of developing diabetes [18-20], increases cardiovascular risk factors [17, 21], causes hair, skin, and nail problems [21, 22], and has complex effects on the immune system [22, 23]. Although it has been suggested that the diabetes-like condition induced by rapamycin might be benevolent, resembling starvation-induced diabetes [24], the complete spectrum of side effects is likely to mask any anti-aging effects in humans, and to have a detrimental effect on lifespan overall. Thus, it is unlikely that rapamycin in its current form would have a beneficial effect in healthy humans, and it remains uncertain whether mTOR signaling could ever be targeted without the development of side effects.

There are two major protein complexes that contain mTOR, mTORC1 and mTORC2 [25]. Although rapamycin has been considered a specific inhibitor of mTORC1, chronic exposure to the drug can also disrupt mTORC2 in some cell lines [26] and *in vivo* [27]. We have previously demonstrated that rapamycin-induced insulin resistance is caused mainly by the “off-target” disruption of mTORC2, and that more specific targeting of mTORC1 using a genetic strategy can extend life without interfering with glucose metabolism [27]. This raises the hope that more specific pharmacological targeting of mTORC1 will be possible, and could replicate the beneficial aspects of rapamycin treatment with fewer negative consequences.

While it remains to be tested whether mTORC1 inhibition *per se* accounts for many of the detrimental effects of rapamycin, it is clear that this complex mediates the drug's effects on mitochondria in mammalian cells. Rapamycin decreases the expression of mitochondrial mRNAs in cultured muscle cells [28, 29] and suppresses oxygen consumption [28, 30, 31]. Decreased mitochondrial respiration is observed even in short-term experiments, suggesting that the effects of rapamycin are mediated in part by a post-translational mechanism. These effects are replicated by loss of mTORC1 function, but not by loss of mTORC2 function [28, 30]. Moreover, mTORC1 binds to the promoters of affected mitochondrial transcripts [29], providing further evidence that mTORC1, and not mTORC2, mediates the mitochondrial effects of rapamycin. These findings raise the possibility that rapamycin-treated mice might become frail and prone to bioenergetic failure, despite having increased longevity. Such effects in the face of mTORC1 inhibition might be considered a trade-off that could compromise survival in the wild, and possibly in humans, but would lead to increased longevity in the protected setting of a mouse colony. Therefore, we tested whether defects in mitochondrial biogenesis and function are apparent in the skeletal muscles of rapamycin-treated mice.

## RESULTS

**Figure 1:**
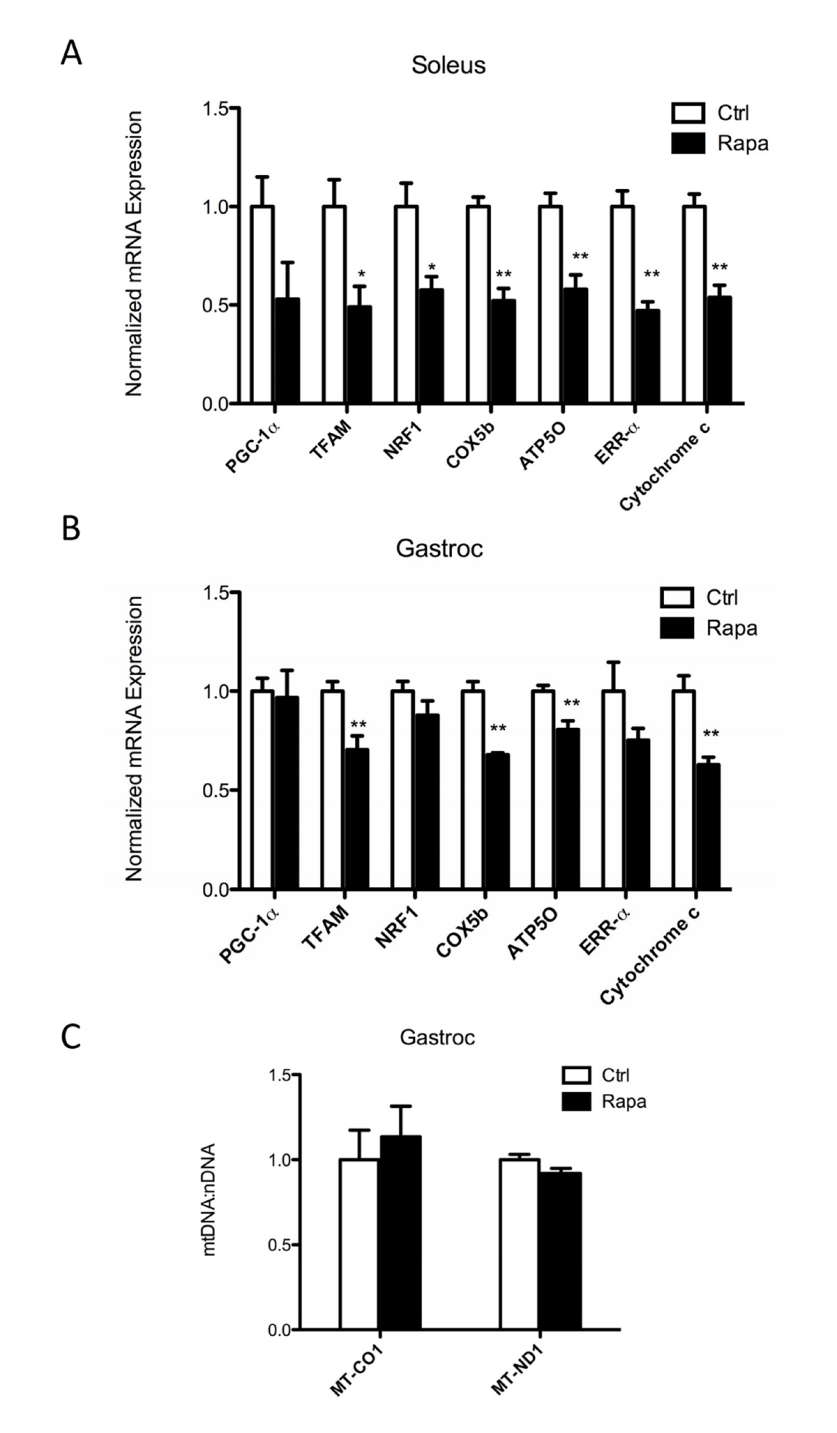
Rapamycin decreases expression of mitochondrial genes in skeletal muscle (**A, B**) Transcript levels for mitochondrial transcription factors (PGC-1α, TFAM, NRF1 and ERRα) and mitochondrial DNA encoded genes (ATP5O, COX5b and cytochrome c) were measured in (**A**) soleus and (B) gastrocnemius (gastroc) muscles following 2 weeks of daily rapamycin treatment. (**C**) Relative mitochondrial DNA copy number was measured in gastrocnemius muscles by determining the ratios of two mtDNA-encoded genes (MT-CO1 and MT-ND1) to the nuclear gene NDUFV1. Data were obtained from C57BL/6 mice following an overnight fast after the last rapamycin injection. Open columns, control; Filled columns, rapamycin. *p<0.05, **p<0.01. Error bars show s.e.m; n=5.

Rapamycin treatment (2 mg/kg daily by intraperitoneal injection) decreased the mRNA expression of genes involved in mitochondrial biogenesis, including mitochondrial transcription factor A (TFAM), nuclear respiratory factor 1 (NRF1), and estrogen-related receptor α (ERRα), as well as genes involved in oxidative phosphorylation, including cytochrome c oxidase subunit 5B (COX5b), ATP synthase subunit O (ATP5O), and cytochrome c in gastrocnemius and soleus muscles, but not in the liver (Figures 1 and S1). These changes were most prominent in the highly oxidative soleus muscle, consistent with the findings of Cunningham et al. [29] and Blattler et al. [32].

**Figure 2:**
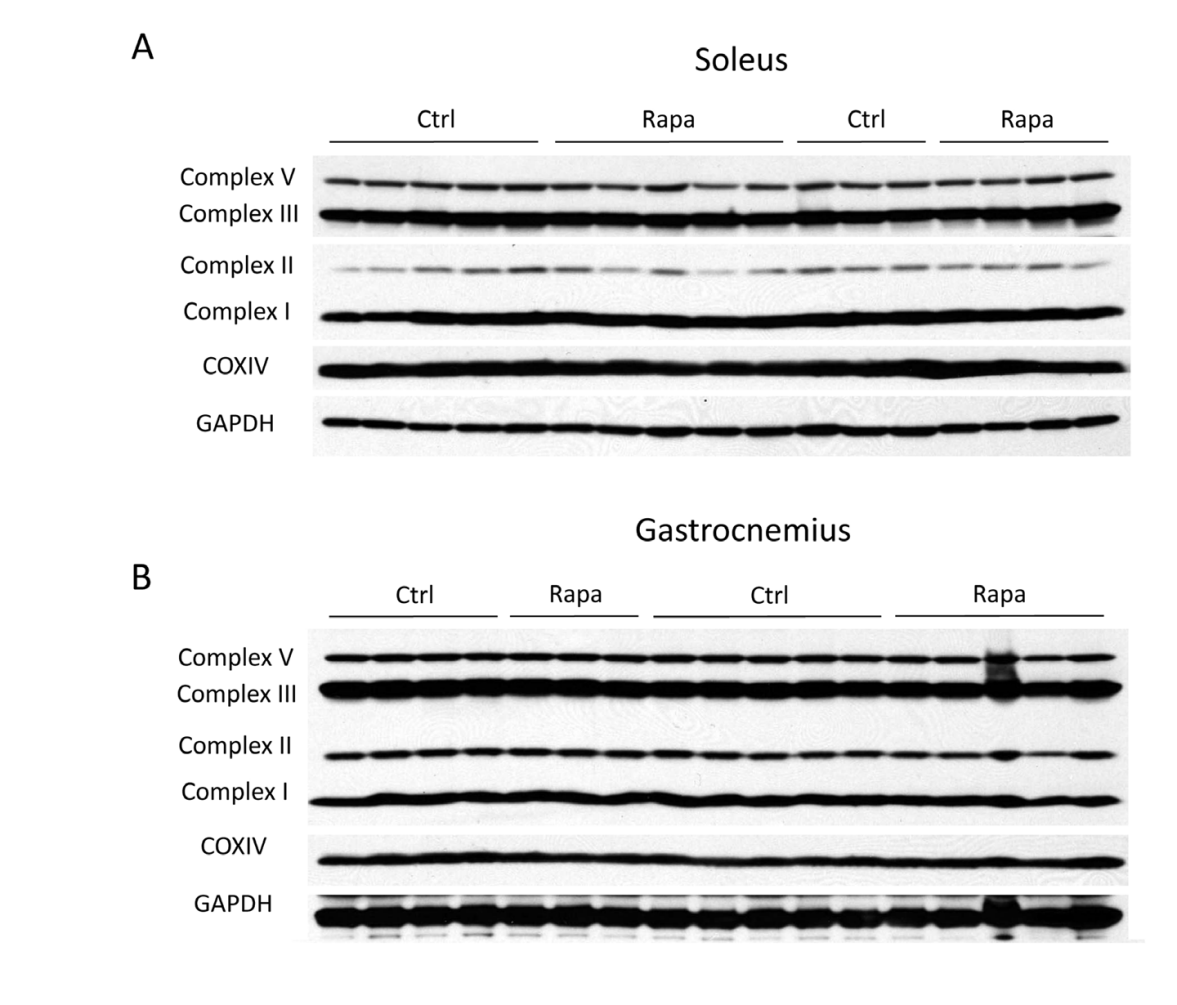
Rapamycin has no major effects on mitochondrial protein levels Representative subunits of each electron transport complex were detected by Western blotting using a cocktail of monoclonal antibodies from MitoSciences. Because the complex IV subunit was not detected using the cocktail, a separate COXIV antibody was also used. The identities of the probed subunits are as follows: complex I - NADH dehydrogenase 1 beta subcomplex 8 (NDUFB8); complex II - succinate dehydrogenase subunit B (SDHB); complex III - ubiquinol-cytochrome c reductase core protein 2 (UQCRC2); complex V -ATP synthase subunit alpha (ATP5A). Proteins were measured in (**A**) soleus or (**B**) gastrocnemius (gastroc) muscle following 2 weeks of rapamycin treatment. Data were obtained from C57BL/6 mice following an overnight fast.

Despite clear changes in message levels, we found that the expression of mitochondrial proteins involved in oxidative phosphorylation was unchanged by rapamycin treatment. We employed a series of monoclonal antibodies that detect representative subunits of each oxidative phosphorylation complex. This approach is predicted to give a reliable indication of overall complex assembly, since the subunits targeted by the monoclonal antibodies are labile when not properly incorporated into their respective oxidative phosphorylation complexes. No consistent changes in mitochondrial protein expression were observed in either the gastrocnemius or soleus muscles (Figure 2), or in the liver (Figure S2). Therefore, expression of mitochondrial proteins in the skeletal muscles of C57BL/6 mice was not affected by two weeks of intraperitoneal injection of rapamycin at a dose sufficient to cause metabolic dysfunction and to extend life.

**Figure 3:**
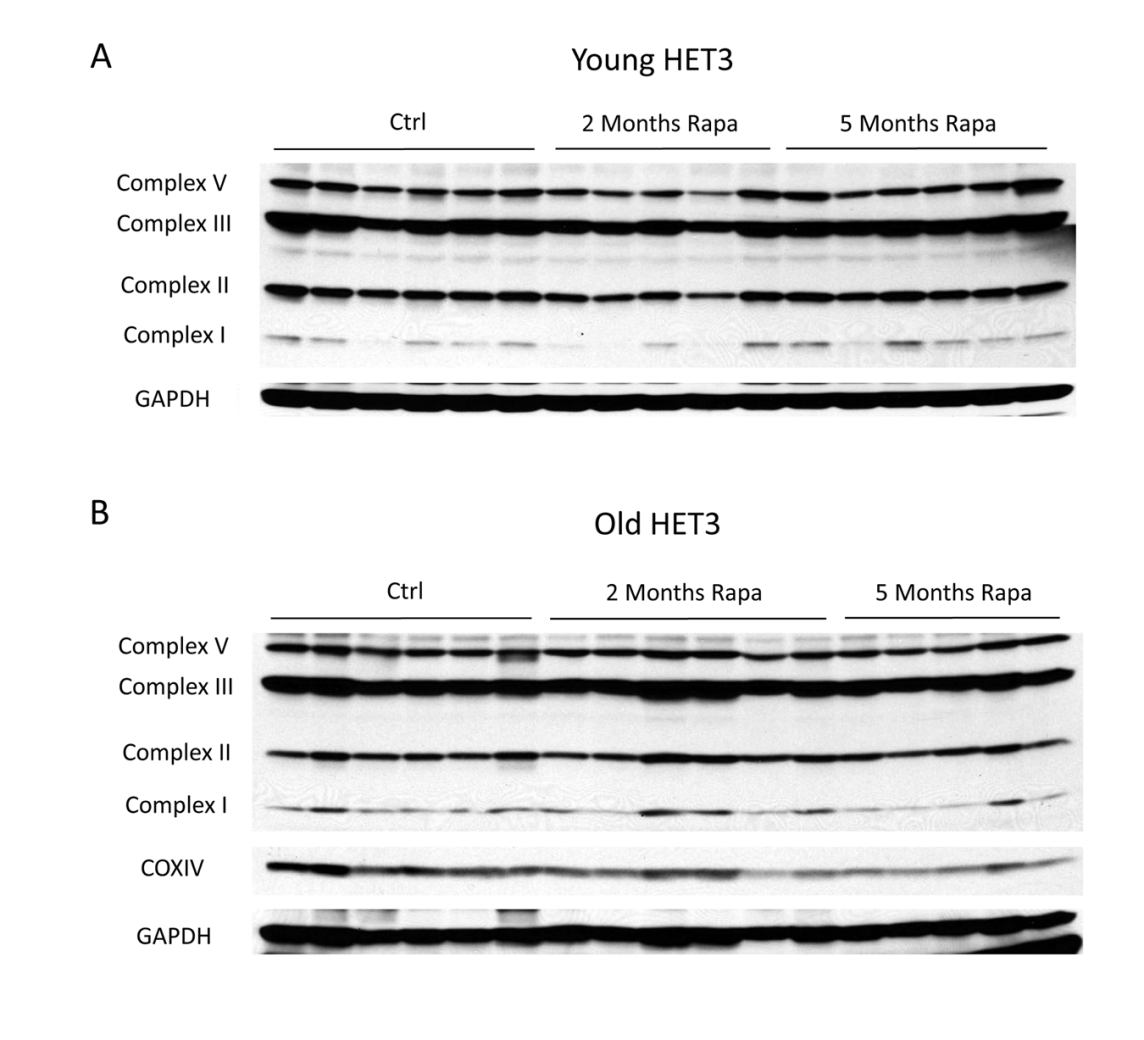
Rapamycin does not change mitochondrial protein expression in Het3 mice from invention testing program Mitochondrial oxidative phosphor-ylation complexes were measured in (**A**) young (6-month-old) or (**B**) old (21-month-old) HET3 mice treated with rapamycin-containing diet for 2 months or 5 months. Antibodies as described for figure 2.

Given the range of dosing strategies that have been used for rapamycin [9], as well as the long half-life of some mitochondrial proteins [33], we chose to examine the expression of mitochondrial proteins under the specific conditions that have been shown to increase longevity. Accordingly, these experiments employed HET3 mice (offspring of a cross between Balb/cByJ × C57BL/6J F1 mothers and C3H/HeJ × DBA/2J F1 fathers), which are expected to be free from any recessive defects present in the parental lines, display a long lifespan, and are known to respond to DR [34, 35]. Young or old HET3 mice were fed a diet containing encapsulated rapamycin at 14 ppm for 2 or 5 months and compared to age-matched controls fed the same diet lacking rapamycin. The effects of this regimen on glucose homeostasis were described previously [36]. As was the case in C57BL/6 mice injected with rapamycin, mitochondrial protein expression was unchanged in HET3 mice fed the rapamycin-containing diet for up to five months (Figure 3). Therefore, we were not able to detect any consistent change in mitochondrial protein expression using the rapamycin treatment regimen that extends mean and maximum lifespan in mice.

**Figure 4:**
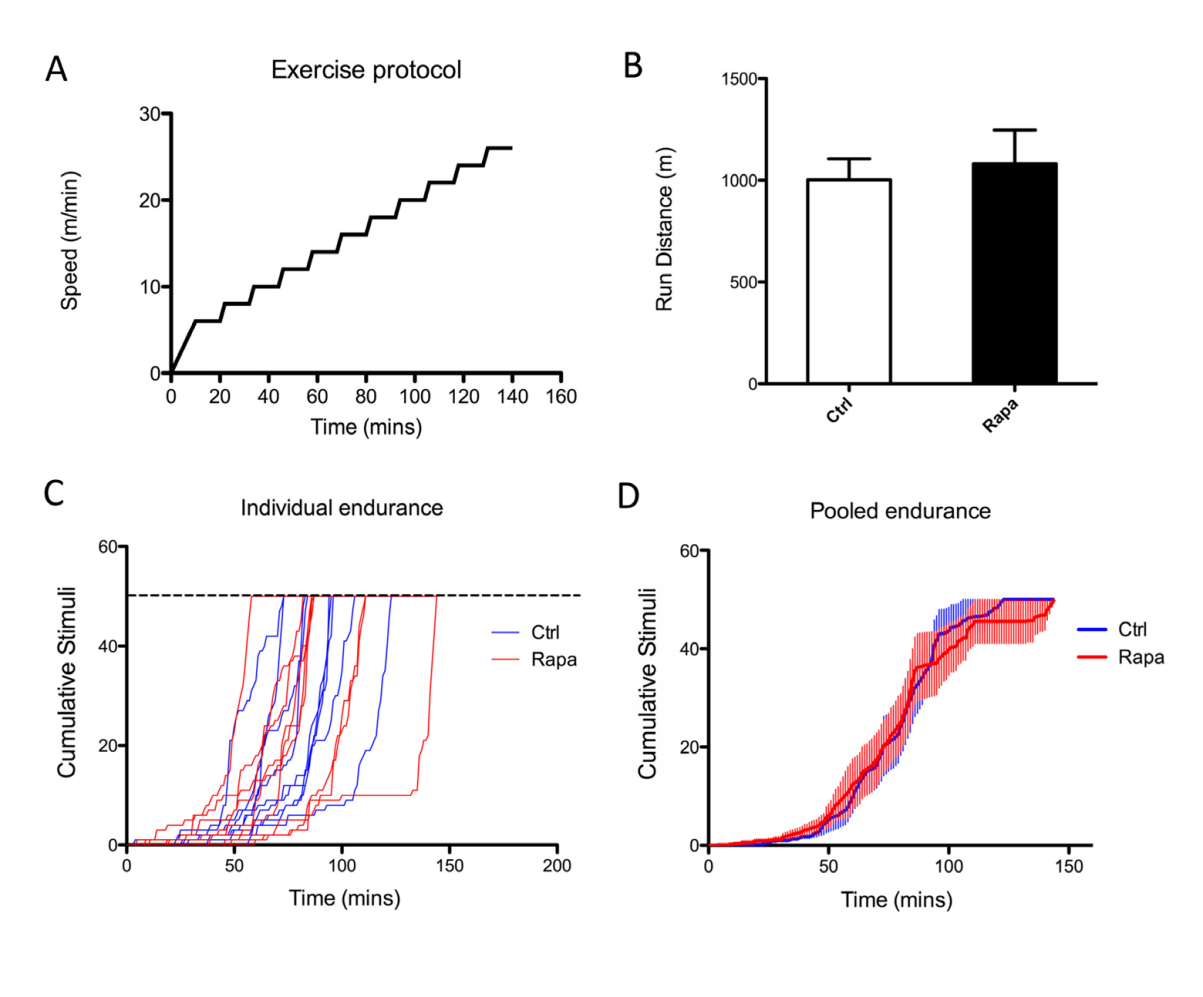
Rapamycin does not affect treadmill endurance 8 week old male C57BL/6 mice were injected with rapamycin (2mg/kg/day) for 2 weeks, then subjected to exercise capacity test. (**A**) Exercise protocol - mice were placed on treadmill and given a 10-minute warm up period, followed by increases in speed at 12-minute intervals. (**B**) Total running distance. Error bars show s.e.m; n=9. (**C, D**) Cumulative shocks for individual (C) or pooled (D) control and rapamycin treated mice.

Our previous studies in cultured myoblasts suggested that rapamycin can impact mitochondrial function even in the absence of changes in protein expression [28], consistent with findings in two previous reports that employed other cell types [30, 31]. To determine whether mitochondrial performance might be impaired in rapamycin-treated animals, we studied treadmill endurance in a second cohort of animals. Rapamycin-treated mice were able to run the same distance as untreated littermates (Figure 4), suggesting that there was no overt deficit in skeletal muscle mitochondrial function.

**Figure 5:**
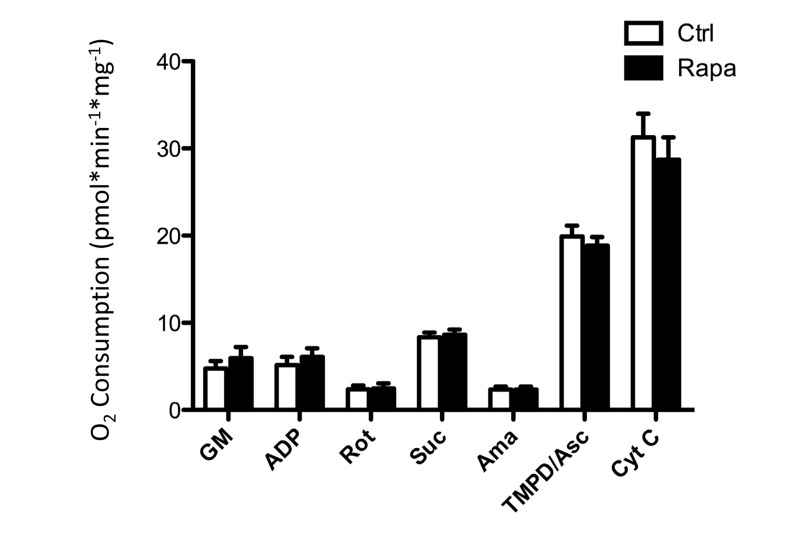
Oxygen consumption in isolated soleus muscles is not affected by prior *in vivo* rapamycin treatment After a one week recovery period, with continued rapamycin dosing, soleus muscle fibers were isolated form the animals represented in figure 4, permeabilized with digitonin, and subjected to respirometry in the presence of mitochondrial substrates and inhibitors using an Oxygraph-2K chamber (Oroboros). GM: Glutamate and Malate; ADP; Rot: Rotenone; Suc: Succinate; Ama: Antimycin A; TMPD/Asc: tetramethylphenylenediamine and ascorbate; Cyt C: Cytochrome c. Error bars show s.e.m; n=5.

**Figure 6:**
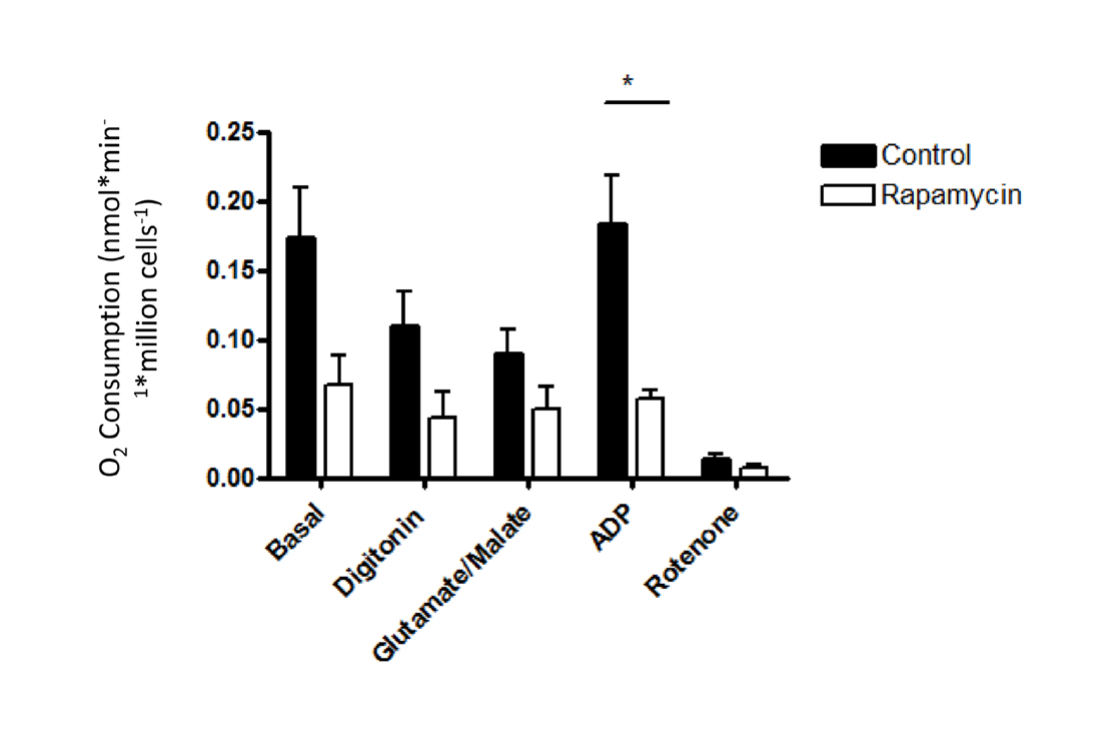
The reduced oxygen consumption of rapamycin-treated cells is maintained following permeabilization Oxygen consumption was measured before and after digitonin-permeabilization in C2C12 myoblasts that had been exposed to 500 nM rapamycin for 24 hr. The following additions were made sequentially: 0.08 mg/ml digitonin, 2 mM glutamate plus 0.4 mM malate, 200 μM ADP, 2 μM rotenone. Rates of oxygen consumption were expressed as nanoatoms of oxygen consumed per minute per million cells using each substrate for three independent experiments. Error bars show s.e.m; n=3. *p<0.05.

To directly test mitochondrial function after rapamycin treatment *in vivo*, oxygen consumption was measured in isolated, permeabilized soleus muscle fibers by high resolution respirometry using an Oroboros Oxygraph 2K. Under all conditions tested, respiration was equivalent in soleus muscles isolated from rapamycin-treated or control mice (Figure 5). Thus, we were not able to detect any consequence of rapamycin treatment on mitochondrial function *in vivo*. This result contrasts somewhat with the findings of Cunningham et al., who were able to detect a decrease in respiration from soleus muscle homogenates following rapamycin treatment, albeit using a slightly higher dose in a different strain [29]. In order to test whether the permeabilization regimen might have masked any differences in oxygen consumption in our experiments, we also studied cultured myoblasts before and after the addition of digitonin. The decrease in oxygen consumption in intact cells that had been pre-treated with rapamycin remained readily apparent following the addition of digitonin and exogenous substrates (Figure 6), indicating that the permeabilization step would not have masked any differences in oxygen consumption between treated and control soleus muscles.

## DISCUSSION

Rapamycin impairs mitochondrial biogenesis and function in cultured muscle cells, which suggests a potential complication of targeting mTOR signaling as a means of promoting longevity in healthy humans. Interventions that promote longevity, including dietary restriction and genetic manipulations, often lead to increased mitochondrial biogenesis, and this has been hypothesized to contribute to the effects on lifespan [37-41]. Moreover, a growing body of evidence supports the concept that mitochondrial “reserve” capacity declines with age and is a major determinant of the ability to overcome stresses [42]. Thus, the inhibition of mitochondrial function by rapamycin despite extending longevity appears somewhat paradoxical. Our results suggest that rapamycin does not decrease mitochondrial protein expression or impair function in skeletal muscle *in vivo*. Although this conclusion resolves the paradox, it is also necessary to note that our findings imply that rapamycin extends life in the absence of any obvious increase in mitochondrial biogenesis, calling into question the concept that tissue mitochondrial content might be limiting for lifespan in mice.

We found that rapamycin clearly decreased the mRNA expression of mitochondrial genes in skeletal muscle, consistent with its effects in cell culture [29], and the observations of Blattler et al. [32]. The ultimate effect on protein levels might be predicted to be even more dramatic, since inhibition of mTORC1 can also have a negative influence on translation [43]. Therefore, it was somewhat surprising to see that mitochondrial protein expression and endurance were not significantly affected. Although these results could be reconciled by reduced turnover of mitochondria in the presence of rapamycin, we do not favor this explanation because inhibition of mTORC1, if anything, should increase degradation of cellular components via autophagy [44]. Indeed, during the preparation of this manuscript, Drake et al. [45] have provided direct evidence that mitochondrial protein turnover in skeletal muscle is unaffected by rapamycin treatment. Another possibility is that decreases in the mRNA abundance for mitochondrial genes are compensated by increased translation. Nuclear-encoded mitochondrial transcripts display distinguishing structural characteristics in the untranslated regions that can contribute to preferential translation under specific conditions, such as dietary restriction [46, 47], and translation of mtDNA-encoded transcripts is subject to many factors that remain incompletely understood [48]. A third possibility is that the assembly of oxidative phosphorylation complexes is better coordinated in the presence of rapamycin, which might prevent the degradation of partial or misfolded complexes [49]. The mechanism by which mitochondrial abundance is maintained in the presence of fewer transcripts will be an interesting area for further investigation.

It is known that total loss of mTORC1 in skeletal muscle, due to deletion of the essential subunit Raptor, or the catalytic subunit mTOR, causes profound mitochondrial dysfunction [50, 51]. Mitochondria are also affected in mice lacking YY1, the transcription factor that recruits mTORC1 to many mitochondrial genes [32]. The fact that rapamycin-treated mice are able to compensate for the reduction in mTORC1 activity suggests a number of possibilities. First, it may be that only a small fraction of normal mTORC1 activity is required to maintain mitochondrial function, and that higher or continuously delivered doses of rapamycin, or more potent mTOR inhibitors, will indeed cause overt mitochondrial dysfunction. In support of this, Cunningham et al detected a reduction in the respiratory capacity of soleus muscle (but not gastrocnemius muscle) using only a slightly higher dose than the present study (2.5 mg/kg in 6-week old Balb/c mice, [29]). Alternatively, mTORC1 activity may be more important for establishing a healthy pool of mitochondria during development than in adult mice, or the rapamycin-resistant functions of mTORC1 [52] may be sufficient to preserve mitochondria. Interestingly, mTORC1 activity is not an absolute requirement for healthy mitochondria, as mitochondrial function can be restored in mice lacking mTOR through the genetic or pharmacological stimulation of mitochondrial biogenesis pathways [53].

In these experiments, we have focused on skeletal muscle because the impairment of mitochondrial biogenesis by rapamycin has been previously demonstrated in myotubes [29], which is consistent with our own findings, and because mTORC1 inactivation has been shown to cause mitochondrial dysfunction in muscle *in vivo* [50, 51]. Although we also observed no significant effects of rapamycin on the expression of mitochondrial components in the liver, we cannot exclude the possibility that rapamycin may impair mitochondrial function in other tissues or cell types. For instance, neurons have very high energetic demands and are reliant on mitochondrial function [54]. Although the effects of rapamycin on neuronal mitochondria have not been measured directly, it is interesting to note that the drug has therapeutic effects in several neurodegenerative disease models [55], which might alleviate concerns that it could be inhibiting bioenergetics. Nevertheless, a comprehen-sive assessment of rapamycin's effects on mitochondria throughout the body is still lacking, and the possibility of impaired mitochondrial function in a specific tissue should be considered carefully in relation to any effects of rapamycin *in vivo*.

Developing therapeutic approaches to target the underlying aging process has the potential to delay or prevent multiple age-related diseases. Inhibition of mTOR signaling with rapamycin has provided some of the strongest evidence to date that this might be possible in mammals. Rapamycin itself is unlikely to have a net benefit in healthy humans due to its side effects, but it remains unclear whether more specific targeting of mTORC1 would alleviate enough of these concerns to constitute a viable strategy. Our present results argue that inhibiting mTORC1 sufficiently to prolong life does not lead to depletion of mitochondrial proteins in skeletal muscle or impair exercise performance in mice. However, many other detrimental side effects of rapamycin have been observed, and some of these may also be mTORC1-dependent. Given the potential benefit of slowing the changes that lead to age-related diseases in humans, understanding and overcoming these side effects should be a high priority for future studies.

## METHODS

### Materials

OXPHOS antibody cocktail (MS604/D1848) was from MitoSciences (Eugene, OR, USA). GAPDH antibody (JC1641540) was from Millipore (Billerica, MA, USA). β-Actin antibody (A5316) was from Sigma-Aldrich (St. Louis, MO, USA). Protease and phosphatase inhibitor cocktail tablets were from Roche (Basel, Switzerland, 11836153001 and 04906845001, respectively). Rapamycin was purchased from Calbiochem (Billerica, MA, USA, 553210). DMEM, fetal bovine serum (FBS), horse serum, insulin, and Trizol were obtained from Invitrogen (Grand Island, NY, USA). Other chemicals were purchased from Sigma (St. Louis, MO, USA) unless noted.

### Animal

Male C57BL/6 mice were obtained from Taconic at approximately 8 weeks of age. Chronic rapamycin treatments were performed by injecting 8-10 week old mice intraperitoneally once daily with rapamycin (2 mg/kg) or vehicle (saline) for 2 or 3 weeks. HET3 mice were produced at the Jackson Laboratory from Balb/cByJ × C57BL/6J F1 mothers and C3H/HeJ × DBA/2J F1 fathers. Young (6 month old) and old (21 month old) mice were fed a rapamycin containing diet or a matched control diet for 2 or 5 months as previously described [36]. The rapamycin diet contains 14 ppm rapamycin encapsulated in chow, from LabDiet®5LG6 (PMI Nutrition International, Bentwood, MO, USA). All experiments were approved by the appropriate Institutional Animal Care and Use Committees and were performed under the supervision of the MIT Department of Comparative Medicine (MIT) or University Laboratory Animal Resources (Penn).

### Quantitative real time RT-PCR assay

Total RNA was extracted using TRIzol reagent. The concentration and purity of RNA were determined by absorbance at 260/280 nm. 1 μg of total RNA was reverse transcribed using a high-capacity cDNA reverse transcription kit (Applied Biosystems, Grand Island, NY, USA) according to the manufacturer's instructions. The cDNA was subjected to real time PCR using SYBR Q-PCR master mix (Applied Biosystems). Primer sequences used to produce gene-specific amplicons are as follows: PGC-1α: forward: ACTATGAATCAAGCCACTACAGAC; reverse: TTCATCCCTCTTGAGCCTTTCG, GAPDH: forward: GGTGAAGGTCGGAGTCAACGGA; reverse: GAGGGATCTCGCTCCTGGAAGA, TFAM: forward: AAGACCTCGTTCAGCATATAACATT; reverse: TTTTCCAAGCCTCATTTACAAGC, NRF1: forward: AATGTCCGCAGTGATGTCC; reverse: GCCTGAGTTTGTGTTTGCTG, COX5b: forward: ACCCTAATCTAGTCCCGTCC; reverse: CAGCCAAAACCAGATGACAG, ATPO: forward: TCTCGACAGGTTCGGAGCTT; reverse: AGAGTACAGGGCGGTTGCATA, ERR-α: forward: ACTGCCACTGCAGGATGAG; reverse: CACAGCCTCAGCATCTTCAA, Cytochrome c: forward: GGAGGCAAGCATAAGACTGG; reverse: TCCATCAGGGTATCCTCTCC, 36B4: forward: GAAACTGCTGCCTCACATCCG; reverse: GCTGGCACAGTGACCTCACACG. A typical reaction contained 250 nmol/l of forward and reverse primer, 1 μl cDNA and the final reaction volume was 20 μl. The reaction was initiated by preheating at 50°C for 2 min, followed by 95°C for 10 min. Subsequently, 40 amplification cycles were carried out with 15 s denaturation at 95°C and 30s annealing and extension at 60°C. Gene expression was normalized to GAPDH or 36B4.

### mtDNA determination

Muscle tissue was digested with 7.5 μl proteinase K (10 mg/ml) in a 250 μl total volume of proteinase K buffer (100 mM Tris-HCL PH 8.5, 5 mM EDTA, 0.2% SDS, 200 mM NaCl) overnight at 55°C. A further 10 μl proteinase K was added the next day, and the reaction was allowed to proceed for one more hour. 250 μl proteinase K buffer and 170 μl 5M NaCl were added and samples were mixed for 1 min, then centrifuged at maximum speed for 15 minutes at 4°C. Supernatants were collected and 1 ml ethanol was added, after which the tubes were inverted several times to mix. Samples were centrifuged at max speed for 15 minutes at 4°C, supernatants were discarded and the DNA pellet was washed with 70% ethanol. The DNA pellet was air dried and resuspended in 50 μl TE buffer. Primer sequences used to produce mitochondrial (MT) and nuclear specific DNA products for quantification of mtDNA/nuclear DNA ratio are as follows: cytochrome c oxidase I (MT-CO1): forward: TGCTAGCCGCAGGCATTAC; reverse: GGGTGCCCAAAGAATCAGAAC, NADH dehydrogenase 1 (MT-ND1): forward: GTGGCTCATCTACTCCACTGA; reverse: TCGAGCGATCCATAACAATAA, NADH dehydrogenase flavoprotein1 (NDUFV1): forward: CTTCCCCACTGGCCTCAAG; reverse: CCAAAACCCAGTGATCCAGC.

### Western blotting

Tissue samples were homogenized using a Tissuemiser Homogenizer (Fisher Scientific, Waltham, MA, USA) in cold RIPA buffer supplemented with phosphatase inhibitor and protease inhibitor cocktail tablets. Tissue lysates were incubated at 4°C with gentle rocking for 15 minutes, then centrifuged at 12,800 rpm for 15 minutes at 4°C to remove insoluble material. Protein concentration was determined by Bicinchoninic Acid (BCA) Assay (Pierce Biotechnology, Rockford, IL, USA). 20ug proteins were separated by sodium dodecylsulphate-polyacrylamide gel electrophoresis (SDS-PAGE) on 8-16% gradient or 7.5% resolving gels. Some of the animals represented in Figure 2 (the right side of each blot) received an intraperitoneal injection of 0.75U/kg insulin 15 minutes prior to sacrifice in order to assess changes in signaling for a prior study [27]. Since this treatment had no appreciable effect on mitochondrial protein levels, insulin status was not considered in the present analysis.

### Endurance exercise

8-week-old male C57BL/6 mice were injected intraperitoneally with saline or rapamycin (2mg/kg) once daily for two weeks. During the third week of injection, 9 control mice and 9 rapamycin-treated mice were tested for endurance on a treadmill (Columbus Instruments Treadmill 3.2, Columbus, OH, USA). Mice were provided with a 10-minute warm up period where speed incrementally increased to 6 m/min. After the 10-minute warm up, mice were subjected to increased speeds at 12-minute increments. Each cycle consisted of 10 minutes of constant speed, followed by 2 minutes of acceleration to 2 m/min faster. Exhaustion was defined as the time point when a mouse had cumulatively received 50 electric shocks from a grid at the back of the treadmill, a point that has been found to reveal differences in endurance in other systems [56]. The number of shocks, total running time and distance were recorded.

### Muscle fiber oxygen consumption

Fresh soleus muscle was isolated from male C57BL/6 mice treated with either 2 weeks of intraperitoneal rapamycin or saline vehicle. Skeletal muscle tissue (2 mg) was placed in cold biopsy preservation solution (BIOPS) consisting of 50 mM K^+^-MES, 20 mM taurine, 0.5 mM dithiothreitol, 6.56 mM MgCl_2_, 5.77 mM ATP, 15 mM phosphocreatine, 20 mM imidazole (pH 7.1 adjusted with 5 N KOH at 0°C), and 10 mM Ca-EGTA buffer. While in cold BIOPS, connective tissue was removed and the muscle fibers were mechanically separated with fine forceps. Muscle fibers were then permeabilized using saponin (50 μg/ml BIOPS). Muscle fibers were added to ice cold saponin/BIOPS and gently agitated for 30 minutes. Fibers werehen transferred into ice-cold mitochondrial respiration medium (MiRO5) with catalase (280 units/ml) and gently agitated on ice for an additional 10 minutes. MiRO5 consists of 110 mM sucrose, 60 mM K^+^-lactobionate, 0.5 mM EGTA, 3 mM MgCl_2_, 20 mM taurine, 10 mM KH2PO4, 20 mM HEPES adjusted to pH 7.1 with KOH at 37°C; and 1 g/L BSA essentially fatty acid free). After weighing, 1 mg of permeabilized fibers were transferred to anOxygraph-2k chamber (Oroboros Instruments, Austria) and suspended in MiRO5 plus catalase at 37°C. After stabilization (~ 10 minutes), oxygen consumption was measured in real-timeafter adding mitochondrial complex substrates and inhibitors sequentially.. Complex I dependent respiration was measured by add glutamate (10 mM), malate (2 mM) and then ADP (5 mM); Complex II dependent respiration was then determined by adding rotenone (0.5 μM) and succinate (10 mM), and finally complex IV dependent respiration was determined by adding Antimycin A (2.5 μM); tetramethylphenylenediamine (TMPD, 0.5 mM) ascorbate (2 mM) and Cytochrome c (10 μM).

### Cell culture

The C2C12 mouse myoblasts were grown in Dulbecco's modified Eagle's medium supplemented with 10% fetal bovine serum and 1% penicillin/streptomycin. The C2C12 cells were treated with 500 nM rapamycin for 24 hours, which we have previously been shown to inhibit respiration [28].

### Myoblast oxygen consumption

Oxygen consumption by C2C12 myoblasts was measured using a standard oxygen electrode (Strathkelvin Instruments, North Lanarkshire, Scotland) in a magnetically stirred, thermostatically regulated chamber at 30°C. Approximately 500,000 cells were suspended in a total volume of 0.15 ml of mitochondrial respiration medium (MiR05 [57], 110 mM sucrose, 60 mM K^+^-lactobionate, 0.5 mM EGTA, 3 mM MgCl 2, 20 mM taurine, 10 mM KHPO_4_, 20 mM HEPES adjusted to pH 7.1 with KOH at 37°C; and 1 g/l BSA essentially fatty acid free). Oxygen consumption was measured before and after each sequential addition of 0.08 mg/ml digitonin, 2 mM glutamate plus 0.4 mM malate, 200 μM ADP, plus 2 μM rotenone. Rates of substrate oxidation were expressed as nanoatoms of oxygen consumed per minute per million cells.

## SUPPLEMENTAL FIGURES


