# Correction for: Rapamycin doses sufficient to extend lifespan do not compromise muscle mitochondrial content or endurance

**DOI:** 10.18632/aging.102976

**Published:** 2020-04-08

**Authors:** Lan Ye, Anne L. Widlund, Carrie A. Sims, Dudley W. Lamming, Yuxia Guan, James G. Davis, David M. Sabatini, David E. Harrison, Ole Vang, Joseph A. Baur

**Affiliations:** 1 State Key Laboratory of Reproductive Medicine, Nanjing Medical University, Nanjing, China; 2 Institute for Diabetes, Obesity, and Metabolism and Department of Physiology, Perelman School of Medicine, University of Pennsylvania, Philadelphia, PA 19104, USA; 3 Department of Science, Systems and Models, Roskilde University, Roskilde, Denmark; 4 Division of Trauma, Critical Care, and Emergency Surgery, University of Pennsylvania, Philadelphia, PA 19104, USA; 5 Whitehead Institute for Biomedical Research and Massachusetts Institute of Technology, Department of Biology, Cambridge, MA 02142, USA; 6 The Jackson Laboratory, Bar Harbor, ME 04609, USA

**Keywords:** correction

**This article has been corrected: **The authors requested the replacement of Figure 3B. They discovered that a residual band from incomplete stripping was inadvertently presented in place of the GAPDH band. The authors revised Figure 3B.

This correction does not change the content of the publication and does not affect the conclusion of this research.

The corrected Figure 3 is provided below.

**Figure 3:**
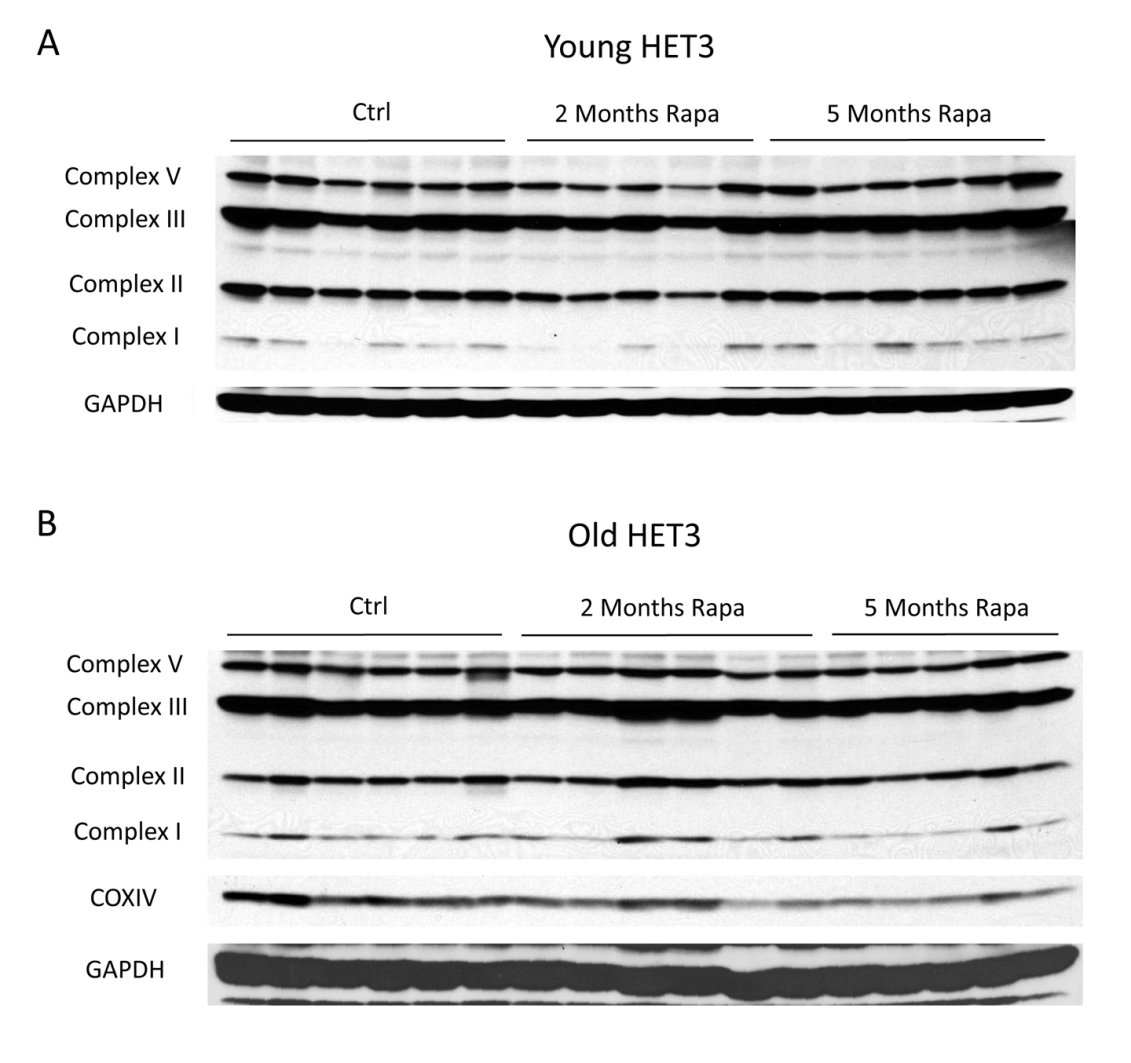
**Rapamycin does not change mitochondrial protein expression in Het3 mice from invention testing program.** Mitochondrial oxidative phosphorylation complexes were measured in (**A**) young (6-month-old) or (**B**) old (21-month-old) HET3 mice treated with rapamycin-containing diet for 2 months or 5 months. Antibodies as described for Figure 2.

Original article: Aging. 2013; 5:539–550.  https://doi.org/10.18632/aging.100576

