# Supplementary material for: Rapamycin doses sufficient to extend lifespan do not compromise muscle mitochondrial content or endurance
Source: Aging (Albany NY). 2013 Jul 16;5(7):539–50. doi: 10.18632/aging.100576 (PMC3765582; doi:10.18632/aging.100576)
Supplement: Supplementary file 1 [file aging-05-539-s001.pdf]

SUPPLEMENTAL FIGURES

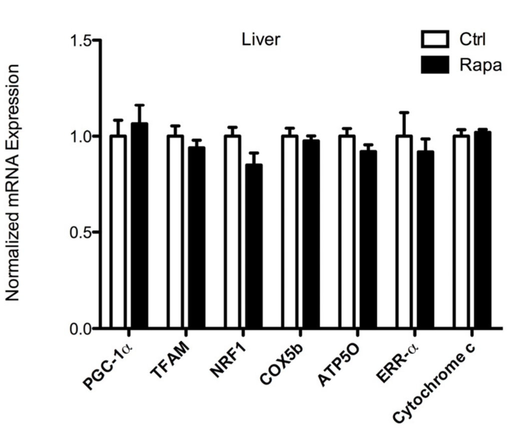

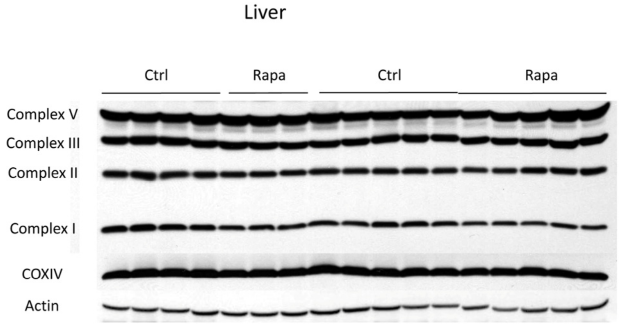

Supplemental Figure S1. Rapamycin does not affect expression of mitochondrial genes in liver. Transcript levels for mitochondrial transcription factors (PGC-1α, TFAM, NRF1 and ERRα) and mitochondrial DNA encoded genes (ATP5O, COX5b and cytochrome c) were measured in liver following 2 weeks of daily rapamycin treatment. Data were obtained from C57BL/6 mice following an overnight fast after the last rapamycin injection. Open columns, control; Filled columns, rapamycin. Error bars show s.e.m; n=5.

Supplemental Figure S2. Rapamycin has no major effects on mitochondrial protein levels in liver. Representative subunits of each electron transport complex were detected by Western blotting using a cocktail of monoclonal antibodies from MitoSciences. Because the complex IV subunit was not detected using the cocktail, a separate COXIV antibody was also used. The identities of the probed subunits are as follows: complex I – NADH dehydrogenase 1 beta subcomplex 8 (NDUFB8); complex II – succinate dehydrogenase subunit B (SDHB); complex III – ubiquinol-cytochrome c reductase core protein 2 (UQCRC2); complex V – ATP synthase subunit alpha (ATP5A). Proteins were measured in liver following 2 weeks of rapamycin treatment. Data were obtained from C57BL/6 mice following an overnight fast.
